# Objective tongue phenotyping identifies phenotypic heterogeneity in diabetic kidney disease: a dual-center clustering analysis

**DOI:** 10.3389/fendo.2026.1873585

**Published:** 2026-07-17

**Authors:** Zhaoxi Dong, Jiayou Liu, Jiyuan Hu, Jiaming Su, Zheyu Xu, Xinhui Yu, Jie Mei, Fengyi Cai, Fawei Li, Xinyue Zang, Runze Wang, Yuanhao Chen, Dongze Li, Weihong Chen, Qingqing Liu, Chengdong Peng, Yang Shi, Hongfang Liu

**Affiliations:** 1 Dongzhimen Hospital, Beijing University of Chinese Medicine, Beijing, China; 2 Department of Nephrology, Beijing Hospital of Integrated Traditional Chinese and Western Medicine, Beijing, China; 3 School of Computer Science and Information Engineering, Hefei University of Technology, Hefei, Anhui, China; 4 Artificial Intelligence Laboratory, Hefei Yunzhen Information Technology Co., Ltd., Hefei, Anhui, China; 5 Treatment Center of Kidney Disease, The First Affiliated Hospital of Henan University of Chinese Medicine, Zhengzhou, China

**Keywords:** clustering analysis, diabetic kidney disease, k-means, objective tongue diagnosis, tongue features, traditional Chinese medicine syndrome, unsupervised learning

## Abstract

**Objective:**

To identify tongue-phenotype subtypes in patients with diabetic kidney disease (DKD) using unsupervised clustering of quantified tongue features, and to compare tongue characteristics and laboratory profiles across subtypes.

**Methods:**

We enrolled 331 patients with DKD from two hospitals in Beijing. Forty-eight continuous tongue features and 3 ordinal/categorical tongue variables were extracted. Continuous variables were z-score standardized. The optimal number of clusters was determined using the elbow method, silhouette width, and gap statistic. K-means clustering was used as the primary analysis, Ward.D2 hierarchical clustering was applied for robustness validation, and partitioning around medoids (PAM) was additionally used in sensitivity analyses. Agreement between methods was assessed by the adjusted Rand index (ARI) and maximum overlap consistency. Tongue features, 40 laboratory variables, and 7 composite indices were compared between subtypes, with Benjamini-Hochberg (BH) correction applied for multiple testing.

**Results:**

Integrating the elbow method, silhouette width, gap statistic, and interpretability, k = 2 was selected. K-means identified Cluster 1 (n = 108, 32.6%) and Cluster 2 (n = 223, 67.4%), with high concordance to hierarchical clustering (maximum overlap consistency, 92.4%; ARI, 0.715). Thirty-nine of 48 continuous tongue features remained significant after correction. Cluster 2 showed higher brightness, lower saturation, a lower coating ratio, thicker coating, and more tooth marks, whereas Cluster 1 showed darker tongue color, higher saturation, more yellow coating, and milder thickness. Coating thickness grade and tooth marks differed significantly between clusters, but fissures did not. No laboratory or composite index differed significantly after multiple imputation and BH correction.

**Conclusions:**

Objective tongue phenotyping identified two reproducible DKD subtypes. Tongue-based heterogeneity did not overlap with conventional laboratory profiles, suggesting complementary value for precision syndrome differentiation in DKD.

## 1 Introduction

Diabetic kidney disease (DKD) is one of the most common and devastating microvascular complications of diabetes and has become the leading cause of end-stage renal disease (ESRD) worldwide (1). Epidemiological data indicate that approximately 20%–40% of patients with type 2 diabetes mellitus (T2DM) develop DKD to varying degrees (2). Beyond markedly increasing the risks of cardiovascular events and all-cause mortality, DKD also imposes a substantial economic burden on patients, families, and health-care systems (3). Accordingly, in patients with established DKD, early phenotypic stratification and individualized intervention are critical for slowing disease progression and improving prognosis (4).

However, DKD is highly heterogeneous (5, 6). Even among patients with comparable glycemic control and renal function indices, the pace of pathological progression, spectrum of complications, and treatment responses often differ substantially (7). In recent years, machine-learning approaches, particularly unsupervised clustering, have shown distinct advantages in uncovering heterogeneity in diabetes and its complications. For example, Liu et al. applied the elbow method and silhouette width to determine the optimal number of clusters in 637 patients with T2DM complicated by myocardial ischemia and successfully identified two subtypes that differed significantly in clinical features and single-photon emission computed tomography (SPECT) imaging parameters, offering a new paradigm for precision phenotyping of diabetic cardiovascular complications (8). Using 11 clinical and metabolic variables, Wang et al. classified 2,267 patients with T2DM and chronic complications into four risk strata by K-means clustering and demonstrated significant differences across subtypes in target-organ damage risk and metabolite profiles; notably, the DKD risk in the high-risk subtype was 11.5-fold higher than that in the low-risk subtype (9). Collectively, these studies indicate that data-driven unsupervised learning can transcend conventional predefined grouping strategies and extract clinically meaningful disease subtypes from high-dimensional data, thereby providing a powerful tool for refined DKD stratification.

Tongue diagnosis is a core component of inspection in traditional Chinese medicine (TCM) and reflects the diagnostic principle of inferring internal pathophysiology from external manifestations. In TCM theory, the tongue is externally associated with the spleen and is closely connected to the zang-fu organs through the meridians; accordingly, tongue body and tongue coating features are traditionally interpreted as reflecting the status of qi and blood, pathogenic factors, and body fluids. Thus, changes in tongue appearance may sensitively mirror physiological and pathological evolution (10). Accumulating modern evidence has shown that tongue features can reflect microcirculation, autonomic function, inflammatory status, and metabolic disturbances (11). With rapid advances in computer vision, spectral analysis, and artificial intelligence, objective tongue diagnosis has made substantial progress (12, 13). Through image segmentation, color-space quantification, texture-feature extraction, and deep-learning algorithms, tongue characteristics can now be digitized in a multidimensional and high-throughput manner. In the diabetes field, Li et al. were among the first to combine vector-quantized variational autoencoder (VQ-VAE) feature extraction with K-means clustering to develop a multi-step unsupervised tongue-image classification strategy, demonstrating that tongue features in diabetes possess intrinsic structures that can be recognized by clustering algorithms and thereby providing objective diagnostic evidence for individualized TCM treatment (14). A quantitative study by Zhang et al. further showed that tongue color parameters in patients with diabetes were influenced by interactions between temperature and humidity, highlighting the potential of tongue appearance as a window into environment–metabolism coupling (15). Together, these studies suggest that objective tongue features can capture metabolic derangements and disease heterogeneity in diabetes, and that data-driven phenotyping based on tongue characteristics has important translational potential (16).

Our previous work has preliminarily demonstrated the diagnostic and syndrome-differentiation value of objective tongue features in DKD. In patients with DKD, the atherogenic index of plasma (AIP) was significantly associated with specific objective tongue features, suggesting that damp-heat in the spleen and stomach, phlegm-turbidity obstruction, and related pathogenic processes may underlie the TCM pathogenesis through which elevated AIP contributes to DKD progression (17). In addition, among patients with damp-heat syndrome in DKD at stages G3 to G5, tongue features showed orderly evolution across disease stages: in stage G5, the tongue body became paler, coating brightness increased, and coating thickness decreased, indicating that worsening renal function and the accumulation of dampness with qi–blood deficiency could be captured by objective tongue characteristics (18). Although these findings have established a close link between tongue features and the pathophysiological evolution of DKD, no study to date has directly applied data-driven unsupervised clustering to objective tongue features in a DKD population.

Against this background, we conducted a dual-center cross-sectional study enrolling 331 patients with DKD to investigate whether data-driven tongue phenotyping can reveal clinically meaningful heterogeneity independent of predefined syndrome labels. K-means unsupervised clustering was applied to 48 objective tongue features—spanning tongue color, coating color, coating properties, and tongue-coating texture—to identify tongue subtypes for the first time in a DKD cohort. We then systematically compared tongue features, routine laboratory indices, and composite metabolic indices across subtypes to determine whether tongue-based stratification captures heterogeneity information beyond what conventional biochemical testing provides. Our findings aim to advance the translational application of objective tongue diagnosis toward precision syndrome differentiation and individualized treatment in DKD.

## 2 Methods

### 2.1 Study population

This was a cross-sectional study that consecutively enrolled inpatients and outpatients with clinically diagnosed diabetic kidney disease (DKD) from Beijing University of Chinese Medicine Dongzhimen Hospital and Beijing Hospital of Traditional Chinese Medicine Combined with Western Medicine between May 2023 and March 2026. Of the 331 enrolled patients, 307 (92.8%) were recruited from Dongzhimen Hospital and 24 (7.2%) from Beijing Hospital of Integrated Traditional Chinese and Western Medicine. The study protocol was approved by the Ethics Committee of Dongzhimen Hospital, Beijing University of Chinese Medicine (2021DZMEC-209–06 and 2023DZMEC-209-01), and all participants provided written informed consent. DKD was diagnosed according to the Chinese Guidelines for the Clinical Diagnosis and Treatment of Diabetic Kidney Disease (2021 edition) and the chronic kidney disease (CKD) framework defined in the Kidney Disease: Improving Global Outcomes (KDIGO) 2022 Clinical Practice Guideline for Diabetes Management in CKD, namely, structural or functional kidney abnormalities persisting for more than 3 months, manifested as persistent albuminuria and/or reduced estimated glomerular filtration rate (eGFR) (19). In this study, DKD was diagnosed clinically on the basis of a history of diabetes, albuminuria/proteinuria, eGFR, serum creatinine, and exclusion of other clearly defined nondiabetic kidney diseases. The inclusion criteria were as follows: (1) age 18–90 years, regardless of sex; (2) fulfillment of the diagnostic criteria for DKD; (3) ability to cooperate with tongue image acquisition; and (4) provision of informed consent. The exclusion criteria were as follows: (1) type 1 diabetes; (2) clearly defined nondiabetic kidney diseases, including primary glomerular diseases, latent nephritis, autoimmune diseases and connective tissue disorders, malignancies, and drug-induced secondary renal injury; (3) recent critical illness within the past 6 months, such as malignant hypertension, myocardial infarction, or cerebrovascular accident; (4) severe primary diseases of the respiratory, digestive, or hematologic systems, or the presence of serious infection or psychiatric disorders; (5) pregnancy or lactation; and (6) obvious tongue staining caused by medications, food, or other factors that could interfere with tongue image interpretation.

### 2.2 Tongue image acquisition and feature extraction

Tongue images were acquired using the YZAI-02 Chinese medicine AI tongue imaging system. The device consists of a standard LED ring light, a portable acquisition unit with a multi-curved face-fitting design to prevent ambient light interference, an ultra-high-resolution camera, and an Android-based host unit. Prior to data collection, all operators received standardized training to ensure consistency in the acquisition procedure and minimize operator-dependent error. During image capture, participants were seated upright, faced the imaging device, and placed their chin against the lower edge of the acquisition port. They were instructed to open their mouth naturally, protrude the tongue without tension, keep the tongue tip slightly lowered, and flatten the tongue surface to fully expose the tongue body for 2–3 s before the physician triggered image capture. If image quality was inadequate or tongue exposure was insufficient, the participant rested briefly and the image was reacquired. Each image was synchronously uploaded to the “AI Open Platform for Tongue Diagnosis in Traditional Chinese Medicine” (Chinese invention patent nos. CN202211523344.1, CN202211315242.0, and CN201910568779.X) for analysis.

After color correction, the platform automatically performed tongue body segmentation and separation of tongue coating and tongue body, followed by multidimensional tongue feature extraction. Color features were quantified in the hue-saturation-value (HSV) color space (hue H, range 0–180; saturation S, range 0–255; value V, range 0–255) and the red-green-blue (RGB) color space (range 0–255). Tongue color and coating color features were extracted from four regions: tongue tip, tongue center, tongue root, and tongue side. For each region, the mean H, S, and V values of tongue color and coating color were calculated, together with the global H, S, and V values of the entire tongue. Tongue body features included the presence or absence of tooth marks and fissures. Tongue coating features included coating ratio, coating thickness grade, and coating texture features. Coating texture features included first-, second-, and third-order color moments as well as gray-level co-occurrence matrix features, including correlation (COR), contrast (CON), energy, angular second moment (ASM), inverse difference moment (IDM), entropy, roughness, and gray mean. The final dataset comprised 48 continuous tongue features and 3 categorical tongue variables (1 ordinal and 2 binary variables).

### 2.3 Clinical data collection and composite index construction

Demographic information (sex and age) and contemporaneous laboratory test results were extracted from the electronic medical record system, including complete blood counts, urinalysis, liver and kidney function parameters, lipid profile, electrolytes, glycated hemoglobin, and urinary protein-related indices. Values reported as below the assay detection limit (e.g., CRP <1) were treated as left-censored and recoded as missing before missingness assessment. Among the continuous laboratory variables, those with missingness exceeding 30% (erythrocyte sedimentation rate [ESR], C-reactive protein [CRP], homocysteine [HCY], urine albumin-to-creatinine ratio [UACR], prealbumin [PA], and cystatin C [CysC]) were excluded from the primary analysis. The remaining continuous variables were handled using multiple imputation.

Based on prior literature and clinical relevance, the following composite indices were constructed: triglyceride-glucose index (TyG), atherogenic index of plasma (AIP), neutrophil-to-lymphocyte ratio (NLR), platelet-to-lymphocyte ratio (PLR), systemic immune-inflammation index (SII), prognostic nutritional index (PNI), and hemoglobin-albumin-lymphocyte-platelet score (HALP):

1. TyG = ln[(TG × 88.57) × (Glu × 18)/2] (20);
2. AIP = log10(TG/HDL-C) (21);
3. NLR = NE#/LY# (22);
4. PLR = PLT/LY# (23);
5. SII = PLT × NE#/LY# (24);
6. PNI = ALB + 5 × LY# (25);
7. HALP = HGB × ALB × LY#/PLT (26).

Triglycerides (TG) and glucose (Glu) were measured in mmol/L; high-density lipoprotein cholesterol (HDL-C) was used for AIP calculation. Hemoglobin (HGB) and albumin (ALB) were measured in g/L; absolute lymphocyte count (LY#), absolute neutrophil count (NE#), and platelet count (PLT) were measured in 10^9/L.

### 2.4 Clustering analysis

Unsupervised clustering was performed on 48 continuous tongue features from 331 patients with DKD. All continuous variables were first standardized using z-score normalization (27). Before clustering, candidate k values were evaluated using three complementary methods: the elbow method, average silhouette width, and the gap statistic (28). The elbow method and silhouette width are classical clustering validation approaches; average silhouette width ranges from −1 to 1, with values >0.25 generally considered indicative of a meaningful clustering structure, whereas the gap statistic, proposed by Tibshirani et al., compares within-cluster dispersion with the expected dispersion under a reference null distribution. To further assess the robustness of the clustering structure, Ward.D2 hierarchical clustering was used as a sensitivity analysis, and the agreement between K-means and hierarchical clustering was evaluated using the adjusted Rand index (ARI; ranging from −1 to 1, with values closer to 1 indicating stronger-than-chance agreement between the two partitioning methods), whereas the maximum overlap consistency percentage was used as a descriptive contingency-table-based measure to quantify the proportion of patients assigned to concordant clusters after optimal label permutation (29–31). Ultimately, considering statistical metrics, clustering stability, and clinical interpretability, k = 2 was selected as the primary clustering solution. To further assess robustness, sensitivity analyses were conducted for k = 3 and k = 5, and partitioning around medoids (PAM) was used as an alternative clustering method. K-means clustering with Euclidean distance, 50 independent random initializations, a maximum of 100 iterations, and minimization of the total within-cluster sum of squares was used as the primary analysis to assign patients to two tongue phenotypes. Using multiple random initializations is a standard strategy for mitigating the risk that the algorithm converges to a suboptimal local minimum—a concern that becomes more relevant in higher-dimensional feature spaces where the initialization of cluster centroids has greater influence on the final partition. Using 50 random starts reduces sensitivity to centroid initialization and increases the chance of obtaining a stable low-WSS solution. To ensure reproducibility, K-means clustering and all analyses involving random sampling were performed with a fixed random seed. Principal component analysis (PCA) score plots of the first two principal components were generated as a linear dimensionality reduction visualization of cluster separation (32). To complement the PCA visualization and assess whether the identified clusters also reflect non-linear structure in the data, t-distributed stochastic neighbor embedding (t-SNE; perplexity = 30, maximum iterations = 1,000) and uniform manifold approximation and projection (UMAP; default parameters) were additionally applied to the same z-score-standardized 48-dimensional feature matrix, with a fixed random seed for reproducibility. These non-linear projections are presented in **Supplementary Figure 4**. Individual silhouette plots were used to illustrate each sample’s fit within its assigned cluster. All analyses and visualizations were performed in R version 4.4.1 using relevant packages including factoextra, cluster, mclust, mice, dplyr, ggplot2, openxlsx, and related packages.

### 2.5 Missing data handling

For laboratory variables with missingness ≤30%, multiple imputation was performed using the MICE (multiple imputation by chained equations) framework (33). MICE is a standard chained-equations approach for handling multivariate missing data and supports automatic pooling of imputed results. Continuous variables were imputed using predictive mean matching (pmm), with clustering group, sex, and other available clinical variables included as predictors. Twenty imputed datasets were generated, each with 10 iterations. Rubin’s rules were then applied to pool the results across the 20 imputed datasets, yielding combined estimates and standard errors.

### 2.6 Statistical analysis

Continuous variables are presented as mean ± standard deviation, and categorical variables as number (percentage). Between-cluster comparisons of continuous tongue features were conducted using linear regression models with clustering group as the independent variable and each tongue feature as the dependent variable, yielding unstandardized regression coefficients (β), 95% confidence intervals (CI), and raw P values; these are fully tabulated in **Supplementary Table 3**. Given the sample size, normality was not formally tested, and linear regression was considered robust for the between-cluster comparisons. P values for all 48 continuous tongue features were adjusted using the Benjamini-Hochberg (BH) procedure to control the false discovery rate (FDR), and adjusted P values (P_adj) < 0.05 were considered statistically significant. Coating thickness grade, an ordinal variable, was compared between clusters using the Wilcoxon rank-sum test, with the W statistic and P value reported; the presence of tooth marks and fissures, both binary variables, was compared using Pearson’s chi-square test (with the χ² statistic and P value reported) or Fisher’s exact test when expected cell counts were <5 (with the exact P value reported). For each laboratory variable and composite index, a linear model was fitted with the variable as the dependent outcome and clustering group as the independent variable. Rubin’s rules were then applied to pool the effect estimates, 95% confidence intervals, and P values. To control for multiple comparisons, BH correction was also applied to the between-group comparisons of laboratory and composite indices. Sex distribution differences were assessed using the Pearson chi-square test or Fisher’s exact test, as appropriate. Between-cluster differences in age were assessed using the independent-samples t-test. Unless otherwise specified, all statistical tests were two-tailed, and raw P < 0.05 was considered nominally significant. Adjusted P values (P_adj) < 0.05 were used as the threshold for statistical significance after multiple-comparison correction. All statistical analyses were performed in R version 4.4.1 (R Foundation for Statistical Computing, Vienna, Austria).

## 3 Results

### 3.1 Clustering analysis

**Figure 1:**
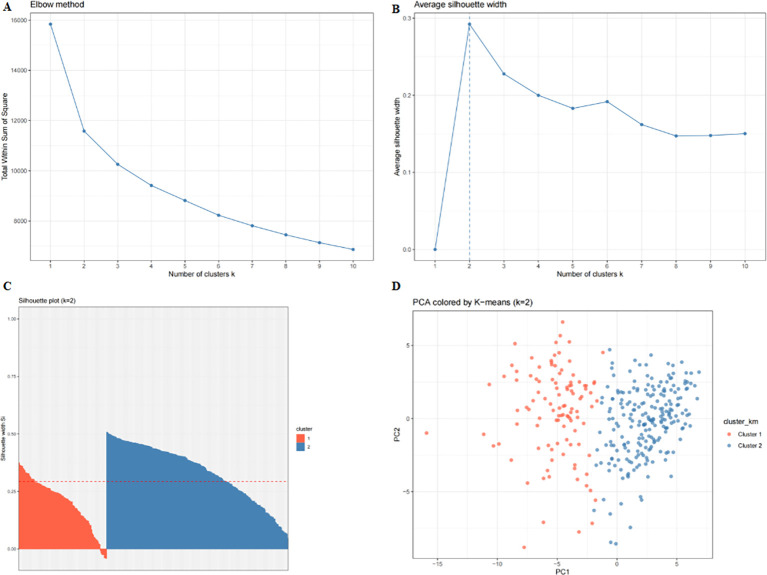
Identification of two reproducible tongue phenotypes in DKD by unsupervised clustering. **(A)** Elbow method based on the total within-cluster sum of squares. **(B)** Average silhouette width across candidate values of k. **(C)** Individual silhouette plot for the K-means solution at k = 2. **(D)** PCA score plot of the first two principal components, colored by K-means cluster assignment. All continuous tongue features were z-score standardized before clustering. The average silhouette width at k = 2 was 0.292. The first two principal components explained 51.3% of the total variance.

In the 331 patients with DKD, z-score-normalized clustering of the 48 continuous tongue features was performed, and the optimal number of clusters was evaluated using the elbow method, average silhouette width, and gap statistic. The elbow plot showed that the decline in total within-cluster sum of squares (WSS) became markedly attenuated after k = 2, suggesting that k = 2 was a reasonable choice (**Figure 1A**). The average silhouette width reached its maximum at k = 2 (0.292) and declined progressively as k increased (**Figure 1B**). According to the Tibshirani one-standard-error criterion, the gap statistic suggested k = 5; however, because the clusters beyond k = 2 contained relatively small sample sizes and were difficult to interpret clinically, and because k = 2 was also supported by the highest silhouette width and the elbow point, k = 2 was ultimately selected as the clustering solution. Using K-means clustering with Euclidean distance, 50 random initializations, and a maximum of 100 iterations, we obtained Cluster 1 (n = 108, 32.6%) and Cluster 2 (n = 223, 67.4%). When the same dataset was partitioned into two clusters using Ward.D2 hierarchical clustering, the results showed high concordance with the K-means assignments (maximum overlap consistency, 92.4%; ARI, 0.715). The average silhouette width at k = 2 was 0.292, and the individual silhouette plot showed that most samples had positive silhouette widths, indicating a stable clustering structure with acceptable within-cluster cohesion (**Figure 1C**). PCA of the first two principal components explained 51.3% of the total variance, and the score plot showed a modest degree of separation between the two clusters along PC1, with some overlap remaining (**Figure 1D**). Complementary t-SNE and UMAP projections of the same standardized feature matrix are provided in **Supplementary Figure 4**; both non-linear embeddings yielded a consistent separation between Cluster 1 and Cluster 2, corroborating the two-cluster structure identified by K-means in the full 48-dimensional space. Sensitivity analyses further supported the primary two-cluster solution. When k = 3 and k = 5 were imposed, the average silhouette widths decreased to 0.2278 and 0.1830, respectively, and cross-method agreement became weaker (maximum overlap consistency: 77.6% and 80.4%; ARI: 0.5325 and 0.5881, respectively), compared with k = 2 (average silhouette width: 0.2923; maximum overlap consistency: 92.4%; ARI: 0.7151). PAM reproduced a nearly identical two-cluster partition (cluster sizes, 224 and 107; average silhouette width, 0.2911; ARI versus K-means, 0.9394). Taken together, these results indicate that the k = 2 K-means solution was the most coherent and reproducible partition in this dataset.

### 3.2 Differences in tongue features between the two clusters

**Figure 2:**
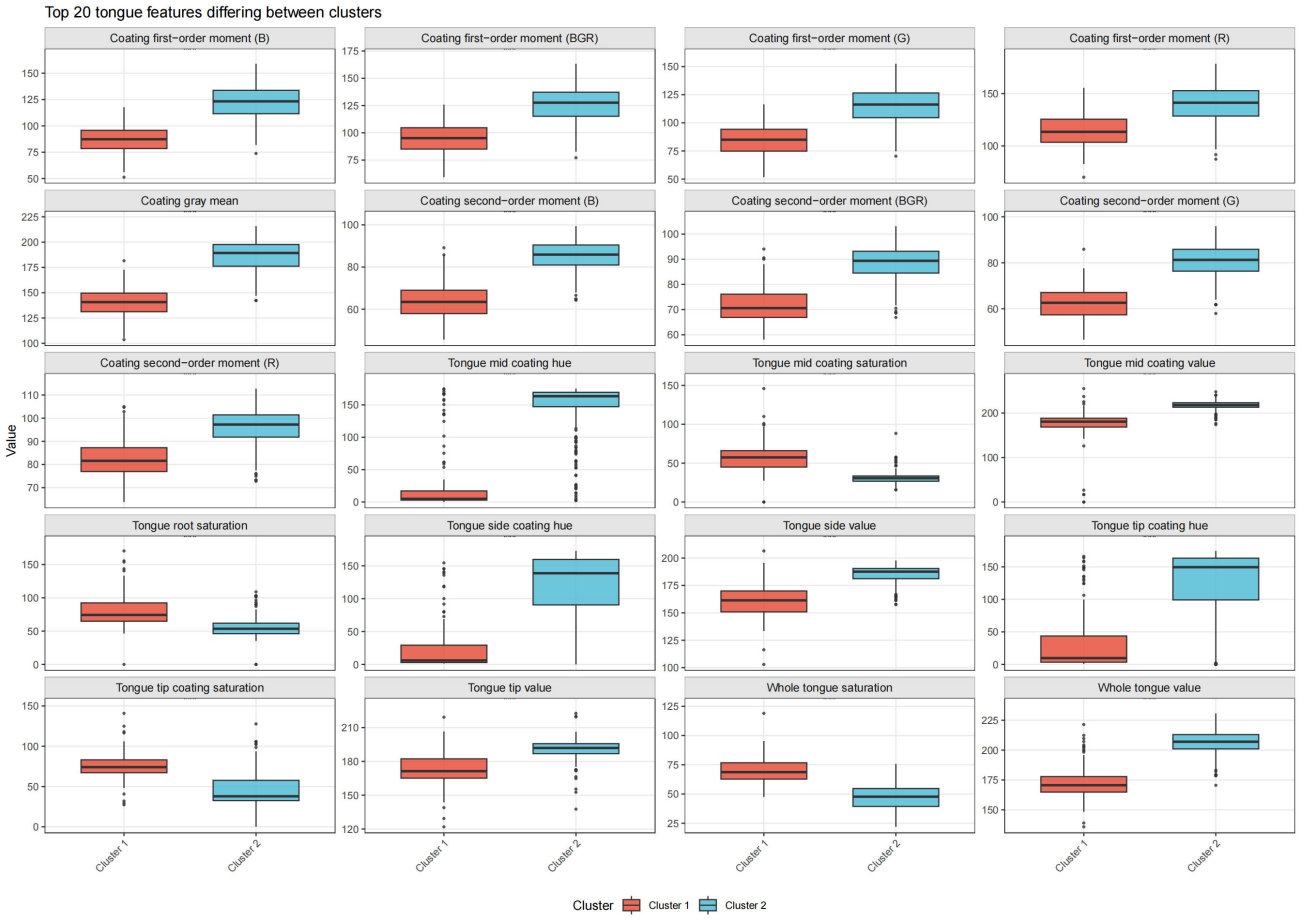
Top 20 continuous tongue features with the smallest BH-adjusted P values between the two clusters. Features are ordered by ascending P_adj. Box-and-whisker plots show the median, interquartile range, and 1.5 × IQR. Significance symbols indicate BH-adjusted P values. Cluster 1 is shown in red and Cluster 2 in blue. The complete comparison of all 48 continuous tongue features is provided in **Supplementary Table 3**.

Among all 48 continuous tongue features, 39 remained significantly different after BH correction (P_adj < 0.05). The 20 most significant features are shown in **Figure 2**.

Overall, Cluster 2 showed a composite tongue phenotype characterized by paler tongue coloration, increased brightness, reduced saturation, a lower tongue-coating ratio, and thicker tongue coating. Specifically, the whole-tongue V value (brightness) was significantly higher in Cluster 2 than in Cluster 1 (P_adj = 9.25 × 10^-72), whereas the whole-tongue S value (saturation) was significantly lower (P_adj = 8.43 × 10^-50). In addition, the coating hue (H) values of the tongue tip, center, root, and side were all markedly higher in Cluster 2 (P_adj ranging from 1.55 × 10^-36 to 1.27 × 10^-55), indicating a tongue coating color shifted toward a paler white/yellow direction. Consistently, the coating gray mean was also significantly higher in Cluster 2 than in Cluster 1 (P_adj = 4.03 × 10^-81). With regard to coating properties, the distribution of coating thickness grade differed significantly between the two clusters (Wilcoxon rank-sum test, P = 3.25 × 10^-15): Cluster 1 was dominated by thin and mildly thick coating, whereas moderate-to-severe coating accounted for 62.3% of Cluster 2 (**Table 1**). The prevalence of tooth marks was significantly higher in Cluster 2 than in Cluster 1 (67.7% vs. 55.6%, P = 0.031), whereas fissure prevalence did not differ significantly between the two clusters (P = 0.197). Tongue-coating texture features, including color moments, correlation, contrast, entropy, and roughness, also differed broadly between the clusters. In Cluster 2, the first- and second-order color moments were significantly higher, whereas the third-order moments shifted toward negative values, suggesting a more homogeneous internal color distribution with increased textural contrast.

**Table 1:** Demographic and categorical tongue characteristics of the two clusters.

Section	Feature	Level	Total	Cluster 1	Cluster 2	Statistic	P value
Demographics	Age (years)		61.9 ± 12.1	61.8 ± 12.3	62.0 ± 12.1	t = -0.126	0.900
Demographics	Sex	Male	228 (68.9%)	73 (67.6%)	155 (69.5%)	χ² = 0.124	0.724
Demographics	Sex	Female	103 (31.1%)	35 (32.4%)	68 (30.5%)		
Categorical tongue features	Coating thickness grade	Thin (0)	22 (6.6%)	17 (15.7%)	5 (2.2%)	W = 6001.5	<0.001
Categorical tongue features	Coating thickness grade	Mildly thick (1)	148 (44.7%)	69 (63.9%)	79 (35.4%)		
Categorical tongue features	Coating thickness grade	Moderately thick (2)	80 (24.2%)	17 (15.7%)	63 (28.3%)		
Categorical tongue features	Coating thickness grade	Severely thick (3)	81 (24.5%)	5 (4.6%)	76 (34.1%)		
Categorical tongue features	Tooth-mark presence	Absent	120 (36.3%)	48 (44.4%)	72 (32.3%)	χ² = 4.653	0.031
Categorical tongue features	Tooth-mark presence	Present	211 (63.7%)	60 (55.6%)	151 (67.7%)		
Categorical tongue features	Fissure presence	Absent	249 (75.2%)	86 (79.6%)	163 (73.1%)	χ² = 1.668	0.197
Categorical tongue features	Fissure presence	Present	82 (24.8%)	22 (20.4%)	60 (26.9%)		

Data are presented as mean ± SD or n (%). Age was compared using the independent-samples t-test, with the t statistic reported. Sex and the binary tongue features were compared using Pearson’s chi-square test or Fisher’s exact test, as appropriate, with the chi-square statistic reported when applicable. Coating thickness grade, an ordinal variable, was compared using the Wilcoxon rank-sum test, with the W statistic reported.

Overall, these results indicate a marked divergence in objective tongue phenotypes between the two clusters. Cluster 2 was closer to a phenotype characterized by “pale tongue, thick white coating, and frequent tooth marks,” whereas Cluster 1 was closer to a phenotype characterized by “darker tongue color, higher saturation, and thinner or mildly thick coating.”

### 3.3 Between-cluster comparisons of laboratory and composite indices

After excluding variables with >30% missingness, the remaining 40 laboratory indices and 7 composite indices were compared between Cluster 1 (n = 108) and Cluster 2 (n = 223). Age and sex distributions were comparable between the two clusters (**Table 1**).

Following multiple imputation across 20 imputed datasets, linear-model fitting, and pooling of estimates using Rubin’s rules, none of the 40 laboratory variables or 7 composite indices differed significantly after BH correction (all P_adj ≥ 0.615; selected results are shown in **Table 2**; complete results for all 47 variables are provided in **Supplementary Table 4**). At the nominal P-value level, only a few variables showed weak trends toward group differences; however, none remained significant after BH correction.

**Table 2:** Comparison of selected laboratory and composite indices between the two clusters (pooled estimates after multiple imputation).

Variable	Cluster 1 (mean ± SD)	Cluster 2 (mean ± SD)	β (95% CI)	t statistic	Nominal P	Adjusted P (P_adj)
WBC (10^9/L)	6.50 ± 1.71	7.06 ± 2.36	0.533 (0.028, 1.037)	2.077	0.039	0.615
NE# (10^9/L)	4.27 ± 1.32	4.74 ± 2.03	0.442 (0.019, 0.865)	2.058	0.040	0.615
A/G	1.41 ± 0.35	1.32 ± 0.31	-0.093 (-0.181, -0.005)	-2.084	0.038	0.615
eGFR (mL/min/1.73 m²)	40.80 ± 31.14	48.15 ± 33.08	7.348 (-0.139, 14.835)	1.931	0.054	0.615
Serum creatinine (μmol/L)	263.54 ± 229.93	236.25 ± 218.09	-27.291 (-78.492, 23.910)	-1.049	0.295	0.928
TyG	9.32 ± 0.82	9.28 ± 0.90	-0.098 (-0.309, 0.113)	-0.913	0.362	0.928
AIP	0.23 ± 0.28	0.21 ± 0.32	-0.028 (-0.104, 0.047)	-0.737	0.462	0.928
NLR	3.22 ± 1.62	3.56 ± 2.40	0.308 (-0.233, 0.848)	1.121	0.263	0.928
SII	698.04 ± 478.26	768.17 ± 569.85	68.838 (-64.496, 202.172)	1.016	0.310	0.928
PNI	44.87 ± 7.99	45.10 ± 7.41	0.197 (-1.579, 1.974)	0.218	0.827	0.949
HALP	34.36 ± 19.70	41.19 ± 65.23	5.169 (-7.109, 17.448)	0.830	0.408	0.928

Data are presented as mean ± SD from the first imputed dataset. β estimates and 95% confidence intervals were pooled across 20 imputed datasets using Rubin’s rules. Nominal P values were obtained from pooled inference based on linear models fitted in each imputed dataset. The t statistic was derived from the pooled estimate and its standard error. Adjusted P values (P_adj) were corrected using the Benjamini-Hochberg procedure across all 47 laboratory and composite indices tested in the full analysis. WBC, white blood cell count; NE#, absolute neutrophil count; A/G, albumin-to-globulin ratio; eGFR, estimated glomerular filtration rate; Scr, serum creatinine; TyG, triglyceride-glucose index; AIP, atherogenic index of plasma; NLR, neutrophil-to-lymphocyte ratio; SII, systemic immune-inflammation index; PNI, prognostic nutritional index; HALP, hemoglobin-albumin-lymphocyte-platelet score.

In the inflammation/hematology domain, both white blood cell count (WBC) and absolute neutrophil count (NE#) were slightly higher in Cluster 2 than in Cluster 1 (WBC: β = 0.533, nominal P = 0.039; NE#: β = 0.442, nominal P = 0.040), but neither remained significant after correction (P_adj = 0.615). Absolute lymphocyte count, absolute monocyte count, platelet count, and the inflammatory composite indices NLR, PLR, and SII did not differ meaningfully between the two clusters.

In the nutrition/metabolic domain, the albumin-to-globulin ratio (A/G) was slightly lower in Cluster 2 (β = −0.093, nominal P = 0.038; P_adj = 0.615), whereas albumin, prealbumin, and total protein were comparable between groups. The prognostic nutritional index (PNI) and HALP score also showed no significant between-cluster differences.

In the renal function and proteinuria domain, eGFR showed a borderline higher trend in Cluster 2 (β = 7.348 mL/min/1.73 m², nominal P = 0.054; P_adj = 0.615), whereas serum creatinine (Scr) was slightly lower (β = −27.291 μmol/L, nominal P = 0.295). No significant differences were observed for 24-h urinary protein excretion, uric acid, or blood urea nitrogen.

In the glucose-lipid metabolism and insulin resistance domain, fasting glucose, glycated hemoglobin, triglycerides, total cholesterol, low-density lipoprotein cholesterol, and high-density lipoprotein cholesterol were similar between the two clusters. The triglyceride-glucose index (TyG) and atherogenic index of plasma (AIP), which reflect insulin resistance and lipid toxicity, respectively, were also not significantly different between Cluster 1 and Cluster 2 (TyG: β = −0.098, nominal P = 0.362; AIP: β = −0.028, nominal P = 0.462).

## 4 Discussion

In this study, we identified, for the first time, two stable tongue phenotypes in 331 patients with DKD based on 48 objective continuous tongue features using unsupervised clustering. The agreement between the two clustering methods (K-means and Ward.D2 hierarchical clustering) was high (92.4%; ARI = 0.715), and the average silhouette width was 0.292, indicating that this binary structure was robust and reproducible. The two subtypes showed significant between-group differences in 39 of 48 continuous tongue variables after correction, spanning multiple dimensions, including tongue color, coating color, coating properties, and coating texture. In contrast, a comprehensive comparison of 40 routine laboratory indices and 7 composite derived indices (TyG, AIP, NLR, PLR, SII, PNI, and HALP) revealed no statistically significant differences after multiple imputation and BH correction. This striking contrast suggests that the heterogeneity captured by objective tongue phenotyping extends beyond the scope of conventional clinical laboratory testing.

### 4.1 Syndrome implications of the two tongue phenotypes

Based on the differences in tongue features between the two clusters, reasonable inferences can be made regarding their TCM pathological implications.

Cluster 2 (n = 223, 67.4%) was characterized by a phenotype of “pale tongue, thick white coating, and a high prevalence of tooth marks.” Specifically, its whole-tongue V value (brightness) was significantly increased, whereas the whole-tongue S value (saturation) was significantly decreased (both P_adj < 10^-49), suggesting a paler tongue body with greater glossiness. The coating H values in all regions were significantly increased (P_adj < 10^-35), indicating a shift of coating color toward a whiter/light-yellow appearance. The coating gray mean was also markedly elevated (P_adj = 4.03 × 10^-81), with coating thickness predominantly moderate to severe (62.3% in total), and the prevalence of tooth marks reached 67.7%. In traditional Chinese medicine, a pale tongue is generally associated with deficiency of qi and blood or yang deficiency, a thick white coating reflects internal accumulation of cold-dampness or spleen deficiency with dampness predominance, and tooth marks are a classic sign of spleen deficiency, qi weakness, and water-damp retention. Taken together, these findings suggest that this phenotype may be more consistent with a pathogenesis centered on spleen-kidney qi/yang deficiency with internal damp-turbidity accumulation. This can be summarized as a syndrome spectrum dominated by “deficiency.”

By contrast, Cluster 1 (n = 108, 32.6%) showed an overall phenotype of darker tongue color, higher saturation, more yellow coating, and milder coating thickness. Its whole-tongue S value was higher and V value was lower, and the coating H values in all regions were significantly lower than those in Cluster 2, suggesting a more yellow coating color. In Cluster 1, thin and mildly thick coating predominated, and the prevalence of tooth marks was also lower than in Cluster 2 (55.6% vs. 67.7%). A dark tongue and yellow coating are classic manifestations of heat and stasis syndrome, indicating that this subtype may be driven primarily by heat, blood stasis, and excess pathogenic factors, and can therefore be classified as a syndrome pattern dominated by “excess.”

Notably, our previous studies showed that in patients with T2DM and DKD, elevated AIP was significantly associated with a yellow, thick coating and increased saturation. The underlying mechanisms were summarized as damp-heat in the middle jiao and phlegm-turbidity obstruction (17). These findings are consistent with the tongue phenotype of Cluster 1 in the present study, further supporting the possibility that Cluster 1 represents a DKD subtype characterized by more active metabolic inflammation and more pronounced damp-heat and stasis.

### 4.2 Dissociation between tongue phenotyping and laboratory indices

The most noteworthy finding of this study is that, although tongue features diverged markedly between the two subtypes, routine laboratory indices and composite derived indices did not show corresponding differences. Rather than representing a contradiction, this dissociation reflects the complementary and biologically non-redundant nature of tongue-based phenotyping relative to conventional biochemical assessment. Among the 40 original variables and 7 composite indices, only WBC, NE#, A/G, and eGFR showed weak trends at the nominal P-value level (nominal P = 0.038–0.054), and none remained significant after BH correction. Indices previously shown to be closely associated with DKD progression and cardiovascular risk, such as TyG, AIP, NLR, and SII, were also entirely overlapping between the two clusters.

This phenomenon, in which tongue phenotypes differ substantially while laboratory indices do not, may have several implications. First, as an integrative, functional, and macroscopic phenotype, the tongue may capture early differences in the systemic state of patients with DKD before overt shifts in biochemical indicators become apparent. Multiple studies suggest that tongue changes may reflect microvascular and inflammatory states, as well as laboratory and microbiome-related alterations, which are not necessarily captured by single biochemical markers such as creatinine, eGFR, or glycated hemoglobin (HbA1c) (34–37).

Second, commonly used composite indices in clinical practice, such as TyG and AIP, are primarily intended to assess insulin resistance and lipotoxicity. Although these indices are associated with the onset and progression of DKD, they may not directly reflect the biological basis of TCM syndromes such as “qi deficiency with dampness predominance” or “stagnation of heat and stasis.” Moreover, emerging evidence points to novel metabolic biomarkers—distinct from conventional lipid and glucose indices—that may more specifically capture the molecular heterogeneity underlying DKD subtypes (38). The molecular mechanisms underlying tongue-based phenotyping remain incompletely understood and warrant further multi-omics investigation, particularly with respect to inflammatory signaling and gut microbiota-associated metabolites (39–41). From a systems perspective, tongue features may capture a biologically orthogonal axis of DKD heterogeneity—one that reflects downstream effector states in peripheral tissues rather than upstream metabolic signals detectable in blood—and one that conventional laboratory panels are not designed to measure. This interpretation is consistent with emerging multimodal precision management frameworks for DKD, in which digital phenotypes such as tongue and pulse features are conceptualized as complementary rather than redundant to biochemical biomarkers (4).

Third, from a clinical perspective, these results also indicate that the syndrome-differentiation information provided by tongue diagnosis is not replaceable by routine laboratory testing, but rather complements it. Within the framework of TCM syndrome differentiation and treatment, Cluster 2 may be more amenable to therapeutic strategies aimed at tonifying qi, strengthening the spleen, and warming yang to resolve dampness, whereas Cluster 1 may respond better to therapies that clear heat, drain dampness, and promote blood circulation to remove stasis. Although this requires confirmation in prospective interventional studies, our findings already provide objective evidence supporting precision syndrome differentiation in DKD.

### 4.3 Relationship to previous tongue-feature studies in DKD

In recent years, research on objective tongue diagnosis in diabetes and DKD has progressed steadily. Our group previously showed that tongue features in damp-heat syndrome among patients with DKD changed dynamically across disease stages, and that AIP was associated with specific tongue features (17, 18). However, these earlier studies all followed a paradigm of “predefined syndrome or index grouping followed by tongue comparison.” In contrast, the present study is the first to apply a data-driven unsupervised clustering approach directly to objective tongue features in a DKD population, thereby avoiding the subjectivity inherent in preassigned syndrome labels. Our findings not only support the feasibility of using tongue features as a tool for phenotyping DKD heterogeneity, but also provide a modern objective basis for the TCM principle of syndrome differentiation and treatment.

### 4.4 Clinical and research implications

The present findings may have several implications for future clinical practice and mechanistic research in DKD. From a clinical perspective, the identification of two objectively defined tongue phenotypes—a deficiency-dominant pattern (Cluster 2) and an excess-dominant pattern (Cluster 1)—suggests that standardized tongue imaging may eventually serve as a low-cost, non-invasive adjunct for syndrome-based stratification in DKD. If these phenotypes are confirmed in independent longitudinal cohorts, they could help refine individualized TCM treatment selection based on the syndrome patterns described above, potentially enabling more targeted application of deficiency-oriented versus excess-oriented therapeutic strategies. Importantly, the objective nature of the tongue features used in this study may reduce the subjectivity inherent in conventional tongue assessment and facilitate reproducible application in multicenter studies.

From a mechanistic standpoint, the dissociation between tongue phenotypes and conventional laboratory indices underscores the need for multi-omics investigations to elucidate the biological underpinnings of tongue-based heterogeneity. Emerging evidence implicates gut microbiota-derived metabolites, phospholipid remodeling, and epigenetic regulation—including DNA methylation—in the pathogenesis of DKD and in mediating the effects of traditional Chinese medicine interventions (41–43). Integrating tongue phenotype data with metabolomics, microbiome profiling, and epigenomic data in future longitudinal cohorts could help delineate the specific molecular pathways that differentiate these subtypes and identify candidate biomarkers or therapeutic targets. Such integrative approaches are aligned with the emerging multimodal precision management framework for DKD and may ultimately bridge objective tongue diagnosis with actionable molecular medicine (4).

### 4.5 Limitations

This study has several limitations. First, the cross-sectional design precludes inference of the causal temporal relationship between tongue phenotypes and disease progression. Whether Cluster 1 and Cluster 2 represent two stable and independent DKD subtypes or different stages within the same disease spectrum remains to be clarified in longitudinal follow-up studies. Second, all samples were derived from two tertiary hospitals in Beijing, which may introduce geographic and institutional selection bias. The current cohort of 331 patients, while within the range of other recent objective tongue-feature clinical studies (34), represents a discovery-stage, dual-center real-world cohort. The stability of the two-cluster solution was supported by multiple internal validation metrics—including average silhouette width, ARI, and cross-method concordance with PAM and Ward.D2 hierarchical clustering—but external validation in independent, geographically diverse DKD cohorts with larger sample sizes is needed before these findings can be generalized. Third, laboratory variables exhibited varying degrees of missingness. Although established multiple imputation methods were applied to variables with missingness ≤30%, the exclusion of several potentially informative markers, particularly CRP, ESR, HCY, UACR, PA, and CysC, may have reduced the power to detect between-cluster differences in inflammatory status, nutritional status, and renal injury. Fourth, clustering is inherently exploratory and does not yield a unique solution; although k = 2 was supported by multiple validation metrics, the gap statistic suggested the possibility of a more granular partition, and alternative phenotypic structures may also have clinical relevance. Therefore, k = 2 should be interpreted as the primary working solution rather than the only possible phenotypic solution. Future studies should seek to prospectively validate the two-cluster structure in independent cohorts and to test whether it maps onto clinically distinct trajectories of DKD progression or treatment response, which would provide the outcome-anchored evidence needed to confirm its biological and clinical significance. It should also be noted that K-means clustering carries several implicit assumptions that may not be fully satisfied in the present dataset. Specifically, K-means minimizes within-cluster Euclidean distances, which implicitly assumes that true clusters are convex and approximately spherical in the feature space and of roughly comparable size; in the current study, however, the two clusters were markedly asymmetric (n = 108 vs. n = 223), and the residual overlap visible in the dimensionality reduction plots suggests that cluster boundaries may deviate from this idealized geometry. Furthermore, K-means is sensitive to outliers, as extreme feature values can shift cluster centroids and distort assignments. These concerns were partially mitigated by z-score standardization of all features and by cross-method validation with Ward.D2 hierarchical clustering and PAM, both of which reproduced a highly concordant two-cluster partition; nonetheless, future studies might consider density-based algorithms or model-based approaches that relax the assumption of spherical cluster shape. A related consideration concerns the dimensionality of the feature space: with 48 tongue features and 331 patients (a feature-to-sample ratio of approximately 1:7), Euclidean distances may become less discriminative as dimensionality increases—a phenomenon known as the curse of dimensionality. To mitigate the associated sensitivity of K-means to initial centroid placement, 50 independent random initializations were performed, and the solution with the lowest total within-cluster sum of squares was retained. Applying dimensionality reduction prior to clustering—for example, retaining the leading principal components—might be explored in future work as an additional strategy against sparsity effects in high-dimensional tongue feature spaces. Fifth, the primary clustering analysis was restricted to the 48 continuous tongue features; the three categorical tongue variables—coating thickness grade (ordinal), and tooth marks and fissures (binary)—were reserved as *post-hoc* validation descriptors rather than clustering inputs, because standard K-means with Euclidean distance is not directly applicable to categorical or mixed-type data. The between-cluster differences observed for coating thickness grade and tooth-mark prevalence (P < 0.001 and P = 0.031, respectively) provided *post hoc* support for the identified partition even without their inclusion in the clustering step. Future studies wishing to incorporate categorical tongue variables into the clustering process could consider algorithms designed for mixed-type data, such as K-prototypes—which extends K-means to handle continuous and categorical variables simultaneously—or Gower’s dissimilarity coefficient combined with PAM, which are specifically suited to heterogeneous feature types. Sixth, tongue image acquisition depends on a specific device and platform algorithm; therefore, portability and generalizability across different devices require external validation. To address this limitation, future work should prioritize multi-device concordance studies and the development of device-agnostic preprocessing or calibration pipelines, with the goal of establishing a standardized acquisition protocol that enables tongue phenotyping to be reproduced across different clinical settings. In addition, the present study initially relied on PCA—a linear dimensionality reduction method—as the primary visualization tool for displaying cluster structure; as PCA captures only linear variance, it may not fully reflect the non-linear geometry of the 48-dimensional tongue feature space. Supplementary t-SNE and UMAP projections were included as complementary non-linear visualizations of the same standardized 48-feature matrix and showed a broadly similar two-cluster pattern. Finally, the present analysis was conducted on standardized numerical tongue features rather than raw image data. Future studies may explore end-to-end unsupervised deep learning approaches—such as variational autoencoders or self-supervised contrastive learning applied directly to tongue images—which may uncover latent visual patterns not fully captured by predefined feature extraction pipelines.

## 5 Conclusion

In summary, objective tongue phenotyping identified two interpretable and reproducible tongue subtypes in 331 patients with DKD: one characterized by a pale tongue, thick white coating, and a high prevalence of tooth marks, which more closely reflected a TCM pattern of spleen-kidney qi deficiency with damp-turbidity retention and thus a deficiency-dominant state; and another characterized by a dark tongue, yellow coating, and fewer tooth marks, which more closely reflected a syndrome of blood stasis and heat accumulation and thus an excess-dominant state. The lack of significant differences in contemporaneous routine laboratory indices and composite derived markers between the two subtypes suggests that objective tongue phenotyping provides a complementary dimension for assessing DKD heterogeneity, independent of conventional biochemical testing. These findings establish a preliminary foundation for the application of objective tongue diagnosis in precision syndrome differentiation and individualized treatment of DKD, while its clinical utility and biological basis warrant further validation in prospective studies and mechanistic investigations.

## Data Availability

The raw data supporting the conclusions of this article will be made available by the authors, without undue reservation.
