# Supplementary material for: Objective tongue phenotyping identifies phenotypic heterogeneity in diabetic kidney disease: a dual-center clustering analysis
Source: Front Endocrinol (Lausanne). 2026 Jul 17;17:1873585. doi: 10.3389/fendo.2026.1873585 (PMC13423681; doi:10.3389/fendo.2026.1873585)
Supplement: Supplementary file 1 [file Supplementaryfile1.docx]

Supplementary Material

# Supplementary Tables and Figures

## Supplementary Tables

**Supplementary Table 1** Standardized cluster centers of the 48 continuous tongue features (z-scores).

| **Variable** | **Cluster 1 center** | **Cluster 2 center** |
| --- | --- | --- |
| Tongue coating ratio | 0.227986597 | -0.110415034 |
| Tongue tip hue | 0.051034392 | -0.024716208 |
| Tongue tip saturation | 0.351412288 | -0.170190705 |
| Tongue tip value | -0.840851174 | 0.407228371 |
| Tongue tip coating hue | -0.894514431 | 0.433217751 |
| Tongue tip coating saturation | 0.834748432 | -0.404272783 |
| Tongue tip coating value | -0.488705828 | 0.236682643 |
| Tongue mid hue | 0.094878716 | -0.04595023 |
| Tongue mid saturation | 0.66790109 | -0.323467792 |
| Tongue mid value | -0.545396821 | 0.264138371 |
| Tongue mid coating hue | -1.072047678 | 0.519197979 |
| Tongue mid coating saturation | 0.974600852 | -0.472004 |
| Tongue mid coating value | -0.912681845 | 0.44201632 |
| Tongue root hue | -0.51710993 | 0.25043889 |
| Tongue root saturation | 0.763651165 | -0.369840026 |
| Tongue root value | -0.71900923 | 0.348219717 |
| Tongue root coating hue | -0.736135589 | 0.356514097 |
| Tongue root coating saturation | 0.722134659 | -0.349733377 |
| Tongue root coating value | -0.547748846 | 0.265277468 |
| Tongue side hue | -0.297189208 | 0.143930199 |
| Tongue side saturation | 0.620516936 | -0.300519413 |
| Tongue side value | -1.027723493 | 0.497731557 |
| Tongue side coating hue | -1.048218797 | 0.507657534 |
| Tongue side coating saturation | 0.681044348 | -0.329833137 |
| Tongue side coating value | -0.435732542 | 0.211027419 |
| Whole tongue hue | -0.720040194 | 0.348719018 |
| Whole tongue saturation | 1.007589238 | -0.487980438 |
| Whole tongue value | -1.139403401 | 0.551818688 |
| Coating first-order moment (B) | -1.043498893 | 0.505371661 |
| Coating first-order moment (G) | -0.976774268 | 0.473056596 |
| Coating first-order moment (R) | -0.823091716 | 0.398627378 |
| Coating second-order moment (B) | -1.128749159 | 0.546658786 |
| Coating second-order moment (G) | -1.083916298 | 0.52494601 |
| Coating second-order moment (R) | -0.886945996 | 0.429552321 |
| Coating third-order moment (B) | 0.617672938 | -0.299142051 |
| Coating third-order moment (G) | 0.561984251 | -0.272171745 |
| Coating third-order moment (R) | 0.469321934 | -0.227294928 |
| Coating first-order moment (BGR) | -0.962150384 | 0.465974177 |
| Coating second-order moment (BGR) | -1.052308336 | 0.509638118 |
| Coating third-order moment (BGR) | 0.594925046 | -0.288125134 |
| Coating correlation | -0.464250629 | 0.22483887 |
| Coating contrast | -0.731887802 | 0.354456873 |
| Coating energy | 0.061690719 | -0.029877119 |
| Coating ASM | 0.076233397 | -0.03692021 |
| Coating inverse difference moment | 0.055309094 | -0.026786467 |
| Coating entropy | 0.057126114 | -0.027666459 |
| Coating roughness | -0.00193666 | 0.000937934 |
| Coating gray mean | -1.180795943 | 0.5718653 |

Cluster centers are reported on the standardized z-score scale. Positive values indicate above-average levels and negative values indicate below-average levels relative to the overall mean of the entire cohort. Centers were derived from the K-means solution at k = 2 after z-score standardization of all continuous tongue features.

**Supplementary Table 2** Agreement between K-means and Ward.D2 hierarchical clustering.

| **Metric** | **Value** |
| --- | --- |
| K-means cluster 1, n | 108 |
| K-means cluster 2, n | 223 |
| Hierarchical cluster 1, n | 228 |
| Hierarchical cluster 2, n | 103 |
| K-means 1 × Hierarchical 1, n | 15 |
| K-means 1 × Hierarchical 2, n | 93 |
| K-means 2 × Hierarchical 1, n | 213 |
| K-means 2 × Hierarchical 2, n | 10 |
| Maximum overlap consistency (%) | 92.4 |
| Adjusted Rand index (ARI) | 0.715 |
| Average silhouette width (k = 2) | 0.292 |

The 2 × 2 contingency table shows the observed overlap between K-means and Ward.D2 hierarchical clustering assignments. Maximum overlap consistency was calculated after optimal permutation of cluster labels. The adjusted Rand index (ARI) ranges from −1 to 1, with higher values indicating better-than-chance agreement. Average silhouette width was calculated for the K-means solution at k = 2.

**Supplementary Table 3** Complete comparison of the 48 continuous tongue features between the two clusters.

| **Variable** | **Cluster 1 (mean ± SD)** | **Cluster 2 (mean ± SD)** | **β (95% CI)** | **t statistic** | **Nominal P** | **Adjusted P (P_adj)** |
| --- | --- | --- | --- | --- | --- | --- |
| Tongue coating ratio | 33.55 ± 13.57 | 29.13 ± 12.59 | -4.420 (-7.399, -1.442) | -2.919 | 0.004 | 0.004 |
| Tongue tip hue | 171.29 ± 16.43 | 170.46 ± 6.74 | -0.823 (-3.332, 1.686) | -0.646 | 0.519 | 0.530 |
| Tongue tip saturation | 81.21 ± 16.62 | 72.91 ± 14.86 | -8.302 (-11.866, -4.738) | -4.582 | <0.001 | <0.001 |
| Tongue tip value | 172.91 ± 15.24 | 190.68 ± 9.27 | 17.770 (15.105, 20.435) | 13.119 | <0.001 | <0.001 |
| Tongue tip coating hue | 35.60 ± 49.64 | 124.03 ± 53.33 | 88.437 (76.408, 100.465) | 14.463 | <0.001 | <0.001 |
| Tongue tip coating saturation | 75.38 ± 18.15 | 45.35 ± 20.47 | -30.037 (-34.591, -25.482) | -12.975 | <0.001 | <0.001 |
| Tongue tip coating value | 129.38 ± 49.66 | 177.02 ± 66.93 | 47.641 (33.379, 61.903) | 6.571 | <0.001 | <0.001 |
| Tongue mid hue | 171.66 ± 19.52 | 169.34 ± 14.76 | -2.320 (-6.116, 1.477) | -1.202 | 0.230 | 0.263 |
| Tongue mid saturation | 66.60 ± 13.89 | 52.39 ± 12.09 | -14.212 (-17.143, -11.282) | -9.540 | <0.001 | <0.001 |
| Tongue mid value | 181.70 ± 12.42 | 194.71 ± 15.95 | 13.015 (9.580, 16.450) | 7.454 | <0.001 | <0.001 |
| Tongue mid coating hue | 30.65 ± 53.49 | 143.64 ± 43.95 | 112.989 (102.089, 123.889) | 20.392 | <0.001 | <0.001 |
| Tongue mid coating saturation | 56.91 ± 19.81 | 31.28 ± 7.88 | -25.631 (-28.635, -22.628) | -16.788 | <0.001 | <0.001 |
| Tongue mid coating value | 173.81 ± 40.59 | 217.07 ± 10.41 | 43.262 (37.570, 48.953) | 14.954 | <0.001 | <0.001 |
| Tongue root hue | 125.12 ± 59.67 | 160.50 ± 32.11 | 35.375 (25.445, 45.304) | 7.008 | <0.001 | <0.001 |
| Tongue root saturation | 80.66 ± 24.84 | 55.75 ± 14.74 | -24.912 (-29.210, -20.615) | -11.403 | <0.001 | <0.001 |
| Tongue root value | 130.89 ± 30.52 | 166.91 ± 28.62 | 36.025 (29.278, 42.772) | 10.504 | <0.001 | <0.001 |
| Tongue root coating hue | 19.53 ± 39.66 | 95.44 ± 67.29 | 75.913 (62.140, 89.687) | 10.842 | <0.001 | <0.001 |
| Tongue root coating saturation | 73.39 ± 24.29 | 44.83 ± 22.45 | -28.563 (-33.881, -23.244) | -10.565 | <0.001 | <0.001 |
| Tongue root coating value | 132.44 ± 42.02 | 171.37 ± 45.38 | 38.926 (28.705, 49.147) | 7.492 | <0.001 | <0.001 |
| Tongue side hue | 166.46 ± 19.90 | 171.70 ± 3.12 | 5.238 (2.555, 7.921) | 3.840 | <0.001 | <0.001 |
| Tongue side saturation | 76.75 ± 14.83 | 63.72 ± 11.65 | -13.025 (-15.970, -10.080) | -8.700 | <0.001 | <0.001 |
| Tongue side value | 160.45 ± 16.04 | 185.02 ± 7.99 | 24.573 (21.977, 27.169) | 18.619 | <0.001 | <0.001 |
| Tongue side coating hue | 25.14 ± 38.63 | 123.33 ± 45.18 | 98.187 (88.235, 108.140) | 19.407 | <0.001 | <0.001 |
| Tongue side coating saturation | 75.68 ± 21.07 | 48.54 ± 24.82 | -27.139 (-32.596, -21.681) | -9.782 | <0.001 | <0.001 |
| Tongue side coating value | 132.72 ± 38.81 | 166.79 ± 54.93 | 34.063 (22.473, 45.653) | 5.782 | <0.001 | <0.001 |
| Whole tongue hue | 123.15 ± 41.93 | 157.81 ± 17.94 | 34.654 (28.176, 41.131) | 10.524 | <0.001 | <0.001 |
| Whole tongue saturation | 69.94 ± 11.66 | 47.15 ± 10.46 | -22.783 (-25.288, -20.278) | -17.892 | <0.001 | <0.001 |
| Whole tongue value | 173.24 ± 14.63 | 206.30 ± 10.33 | 33.058 (30.314, 35.802) | 23.701 | <0.001 | <0.001 |
| Coating first-order moment (B) | 87.01 ± 13.63 | 122.28 ± 16.54 | 35.269 (31.660, 38.879) | 19.221 | <0.001 | <0.001 |
| Coating first-order moment (G) | 84.50 ± 13.65 | 115.33 ± 16.46 | 30.829 (27.232, 34.427) | 16.858 | <0.001 | <0.001 |
| Coating first-order moment (R) | 114.01 ± 16.61 | 140.33 ± 18.16 | 26.322 (22.246, 30.398) | 12.704 | <0.001 | <0.001 |
| Coating second-order moment (B) | 64.79 ± 8.92 | 85.46 ± 6.92 | 20.669 (18.910, 22.428) | 23.115 | <0.001 | <0.001 |
| Coating second-order moment (G) | 62.78 ± 7.49 | 80.77 ± 7.26 | 17.986 (16.294, 19.678) | 20.914 | <0.001 | <0.001 |
| Coating second-order moment (R) | 82.84 ± 9.03 | 96.40 ± 7.63 | 13.568 (11.697, 15.439) | 14.265 | <0.001 | <0.001 |
| Coating third-order moment (B) | -35.89 ± 33.15 | -65.22 ± 26.64 | -29.329 (-35.998, -22.660) | -8.651 | <0.001 | <0.001 |
| Coating third-order moment (G) | -35.29 ± 31.77 | -60.75 ± 26.19 | -25.462 (-31.949, -18.975) | -7.722 | <0.001 | <0.001 |
| Coating third-order moment (R) | -55.83 ± 35.70 | -78.18 ± 27.44 | -22.355 (-29.360, -15.351) | -6.279 | <0.001 | <0.001 |
| Coating first-order moment (BGR) | 95.17 ± 14.34 | 125.98 ± 16.78 | 30.807 (27.111, 34.503) | 16.397 | <0.001 | <0.001 |
| Coating second-order moment (BGR) | 72.10 ± 7.93 | 88.52 ± 6.75 | 16.414 (14.764, 18.064) | 19.570 | <0.001 | <0.001 |
| Coating third-order moment (BGR) | -32.94 ± 38.89 | -65.00 ± 29.89 | -32.062 (-39.693, -24.431) | -8.265 | <0.001 | <0.001 |
| Coating correlation | 0.94 ± 0.02 | 0.95 ± 0.01 | 0.012 (0.008, 0.015) | 6.203 | <0.001 | <0.001 |
| Coating contrast | 586.52 ± 189.57 | 833.42 ± 198.71 | 246.905 (201.753, 292.057) | 10.757 | <0.001 | <0.001 |
| Coating energy | 0.58 ± 0.11 | 0.57 ± 0.10 | -0.009 (-0.032, 0.014) | -0.781 | 0.436 | 0.475 |
| Coating ASM | 0.35 ± 0.12 | 0.34 ± 0.11 | -0.013 (-0.039, 0.013) | -0.965 | 0.335 | 0.374 |
| Coating inverse difference moment | 7.26 ± 2.08 | 7.11 ± 1.66 | -0.148 (-0.565, 0.269) | -0.700 | 0.485 | 0.506 |
| Coating entropy | 3.46 ± 0.73 | 3.40 ± 0.67 | -0.058 (-0.216, 0.100) | -0.723 | 0.470 | 0.502 |
| Coating roughness | 6.40 ± 1.22 | 6.40 ± 1.09 | 0.003 (-0.258, 0.265) | 0.024 | 0.980 | 0.980 |
| Coating gray mean | 140.35 ± 15.36 | 186.39 ± 14.74 | 46.039 (42.592, 49.486) | 26.278 | <0.001 | <0.001 |

Data are presented as mean ± SD, β (Cluster 2 minus Cluster 1), t statistic, nominal P values, and BH-adjusted P values (P_adj). β values were obtained from linear models with clustering group as the independent variable, and the t statistic was derived from the model coefficient and its standard error. P_adj values were corrected using the Benjamini-Hochberg procedure across all 48 continuous tongue features.

**Supplementary Table 4** Complete comparison of all laboratory and composite indices between the two clusters.

| **Variable** | **Cluster 1 (mean ± SD)** | **Cluster 2 (mean ± SD)** | **β (95% CI)** | **t statistic** | **Nominal P** | **Adjusted P (P_adj)** |
| --- | --- | --- | --- | --- | --- | --- |
| RBP | 45.49 ± 16.31 | 45.17 ± 13.42 | 0.302 (-3.691, 4.295) | 0.149 | 0.881 | 0.964 |
| C1q | 173.45 ± 49.06 | 175.86 ± 49.56 | 2.038 (-10.959, 15.035) | 0.310 | 0.757 | 0.949 |
| HDL-C | 1.14 ± 0.38 | 1.12 ± 0.31 | -0.035 (-0.121, 0.051) | -0.802 | 0.423 | 0.928 |
| 24-h urinary protein | 3502.13 ± 3653.72 | 3365.97 ± 4513.07 | -133.112 (-1154.960, 888.735) | -0.257 | 0.798 | 0.949 |
| HbA1c | 7.91 ± 2.30 | 7.75 ± 2.29 | -0.129 (-0.649, 0.391) | -0.489 | 0.625 | 0.928 |
| CO2CP | 25.05 ± 3.34 | 24.69 ± 3.31 | -0.288 (-1.079, 0.503) | -0.716 | 0.474 | 0.928 |
| pH | 5.76 ± 0.72 | 5.62 ± 0.64 | -0.148 (-0.308, 0.011) | -1.831 | 0.068 | 0.615 |
| A/G | 1.41 ± 0.35 | 1.32 ± 0.31 | -0.093 (-0.181, -0.005) | -2.084 | 0.038 | 0.615 |
| Direct bilirubin | 2.15 ± 1.45 | 2.22 ± 1.55 | 0.043 (-0.331, 0.417) | 0.225 | 0.822 | 0.949 |
| Indirect bilirubin | 7.28 ± 3.90 | 7.69 ± 3.55 | 0.285 (-0.595, 1.165) | 0.637 | 0.524 | 0.928 |
| ALP | 77.75 ± 25.32 | 80.79 ± 41.80 | 4.138 (-4.761, 13.038) | 0.915 | 0.361 | 0.928 |
| Na | 138.95 ± 2.81 | 138.77 ± 3.58 | -0.326 (-1.120, 0.468) | -0.807 | 0.420 | 0.928 |
| Total protein | 64.52 ± 10.22 | 66.00 ± 8.47 | 1.291 (-0.865, 3.448) | 1.179 | 0.239 | 0.928 |
| LDL-C | 2.83 ± 1.47 | 2.68 ± 1.10 | -0.148 (-0.440, 0.144) | -0.998 | 0.319 | 0.928 |
| Total cholesterol | 4.71 ± 2.00 | 4.54 ± 1.42 | -0.204 (-0.587, 0.178) | -1.052 | 0.294 | 0.928 |
| Triglycerides | 2.28 ± 1.88 | 2.27 ± 2.83 | -0.084 (-0.694, 0.527) | -0.269 | 0.788 | 0.949 |
| GGT | 29.24 ± 59.53 | 31.82 ± 36.77 | 1.955 (-9.045, 12.955) | 0.350 | 0.727 | 0.949 |
| Total bilirubin | 9.48 ± 4.83 | 9.80 ± 4.31 | 0.264 (-0.817, 1.345) | 0.480 | 0.632 | 0.928 |
| Calcium | 2.17 ± 0.22 | 2.23 ± 0.22 | 0.047 (-0.007, 0.101) | 1.720 | 0.087 | 0.615 |
| AST | 1.41 ± 0.36 | 1.40 ± 0.40 | 0.003 (-0.089, 0.094) | 0.056 | 0.955 | 0.976 |
| Phosphorus | 19.10 ± 9.51 | 19.63 ± 8.74 | 0.345 (-1.797, 2.486) | 0.317 | 0.752 | 0.949 |
| ALT | 18.35 ± 11.24 | 17.77 ± 12.10 | -0.736 (-3.523, 2.051) | -0.520 | 0.604 | 0.928 |
| MPV | 9.24 ± 1.21 | 9.13 ± 1.38 | -0.096 (-0.407, 0.215) | -0.609 | 0.543 | 0.928 |
| NE# | 4.27 ± 1.32 | 4.74 ± 2.03 | 0.442 (0.019, 0.865) | 2.058 | 0.040 | 0.615 |
| LY# | 1.53 ± 0.68 | 1.61 ± 0.71 | 0.083 (-0.083, 0.248) | 0.982 | 0.327 | 0.928 |
| MO# | 0.45 ± 0.17 | 0.46 ± 0.19 | 0.017 (-0.028, 0.062) | 0.741 | 0.459 | 0.928 |
| NE% | 65.21 ± 8.10 | 66.16 ± 10.14 | 0.593 (-1.630, 2.817) | 0.525 | 0.600 | 0.928 |
| LY% | 23.79 ± 7.59 | 23.54 ± 9.42 | -0.126 (-2.215, 1.962) | -0.119 | 0.905 | 0.967 |
| Albumin | 37.23 ± 7.14 | 37.06 ± 5.74 | -0.216 (-1.682, 1.250) | -0.290 | 0.772 | 0.949 |
| Fasting glucose | 8.39 ± 4.45 | 8.56 ± 4.59 | 0.014 (-1.071, 1.098) | 0.025 | 0.980 | 0.980 |
| K | 4.48 ± 0.56 | 4.39 ± 0.64 | -0.097 (-0.240, 0.046) | -1.331 | 0.184 | 0.928 |
| HCT | 34.95 ± 7.55 | 35.09 ± 6.78 | 0.122 (-1.497, 1.741) | 0.148 | 0.882 | 0.964 |
| PLT | 215.45 ± 71.48 | 220.59 ± 83.94 | 6.084 (-12.545, 24.713) | 0.643 | 0.521 | 0.928 |
| WBC | 6.50 ± 1.71 | 7.06 ± 2.36 | 0.533 (0.028, 1.037) | 2.077 | 0.039 | 0.615 |
| RBC | 3.94 ± 0.87 | 3.93 ± 0.76 | -0.005 (-0.188, 0.177) | -0.059 | 0.953 | 0.976 |
| HGB | 117.39 ± 26.52 | 119.03 ± 22.89 | 1.559 (-4.018, 7.135) | 0.550 | 0.583 | 0.928 |
| Uric acid | 378.00 ± 97.77 | 397.97 ± 108.04 | 21.104 (-3.429, 45.637) | 1.692 | 0.092 | 0.615 |
| BUN | 14.56 ± 8.36 | 13.58 ± 8.62 | -0.994 (-2.962, 0.973) | -0.994 | 0.321 | 0.928 |
| Serum creatinine | 263.54 ± 229.93 | 236.25 ± 218.09 | -27.291 (-78.492, 23.910) | -1.049 | 0.295 | 0.928 |
| eGFR | 40.80 ± 31.14 | 48.15 ± 33.08 | 7.348 (-0.139, 14.835) | 1.931 | 0.054 | 0.615 |
| TyG | 9.32 ± 0.82 | 9.28 ± 0.90 | -0.098 (-0.309, 0.113) | -0.913 | 0.362 | 0.928 |
| AIP | 0.23 ± 0.28 | 0.21 ± 0.32 | -0.028 (-0.104, 0.047) | -0.737 | 0.462 | 0.928 |
| NLR | 3.22 ± 1.62 | 3.56 ± 2.40 | 0.308 (-0.233, 0.848) | 1.121 | 0.263 | 0.928 |
| PLR | 159.38 ± 75.67 | 156.93 ± 78.91 | -2.182 (-21.899, 17.534) | -0.218 | 0.828 | 0.949 |
| SII | 698.04 ± 478.26 | 768.17 ± 569.85 | 68.838 (-64.496, 202.172) | 1.016 | 0.310 | 0.928 |
| PNI | 44.87 ± 7.99 | 45.10 ± 7.41 | 0.197 (-1.579, 1.974) | 0.218 | 0.827 | 0.949 |
| HALP | 34.36 ± 19.70 | 41.19 ± 65.23 | 5.169 (-7.109, 17.448) | 0.830 | 0.408 | 0.928 |

Data are presented as mean ± SD from the first imputed dataset, together with pooled β estimates, 95% confidence intervals, t statistics, nominal P values, and BH-adjusted P values (P_adj). β values were pooled across 20 imputed datasets using Rubin’s rules, and the t statistic was derived from the pooled estimate and its standard error. P_adj values were corrected using the Benjamini-Hochberg procedure across all 47 laboratory and composite indices tested. Variables excluded because of missingness >30% are described in the Methods. Abbreviations are defined in the main text.

**Supplementary Table 5** Sensitivity analysis of clustering solutions for k = 2, 3, and 5.

| **k** | **Kmeans cluster sizes** | **Hierarchical cluster_sizes** | **Maximum overlap consistency (%)** | **ARI** | **Average silhouette width** |
| --- | --- | --- | --- | --- | --- |
| 2 | 1 108 / 2 223 | 1 228 / 2 103 | 92.4 | 0.7151 | 0.2923 |
| 3 | 1 163 / 2 77 / 3 91 | 1 228 / 2 18 / 3 85 | 77.6 | 0.5325 | 0.2278 |
| 5 | 1 28 / 2 79 / 3 23 / 4 89 / 5 112 | 1 103 / 2 48 / 3 77 / 4 18 / 5 85 | 80.4 | 0.5881 | 0.183 |

k, number of clusters. ARI, adjusted Rand index. K-means clustering was performed with Euclidean distance, 50 random initializations, and a maximum of 100 iterations. "Hierarchical cluster sizes" refers to Ward.D2 hierarchical clustering applied to the same z-score-standardized 48 continuous tongue features using Euclidean distance. Maximum overlap consistency was calculated after optimal label permutation of K-means and hierarchical cluster assignments. The k = 2 row reports values from the primary analysis described in the main text.

**Supplementary Table 6** Comparison of K-means and PAM clustering.

| **Method** | **Cluster sizes** | **Average silhouette width** | **ARI versus K-means** |
| --- | --- | --- | --- |
| K-means | 1 108 / 2 223 | 0.2923 | 0.9394 |
| PAM | 1 224 / 2 107 | 0.2911 |  |

ARI, adjusted Rand index. K-means was performed as described in the Methods. PAM (partitioning around medoids) was applied to the Euclidean distance matrix of the same z-score-standardized features at k = 2. Average silhouette widths were computed from each method's cluster assignments. ARI was calculated between K-means and PAM assignments.

## Supplementary Figures


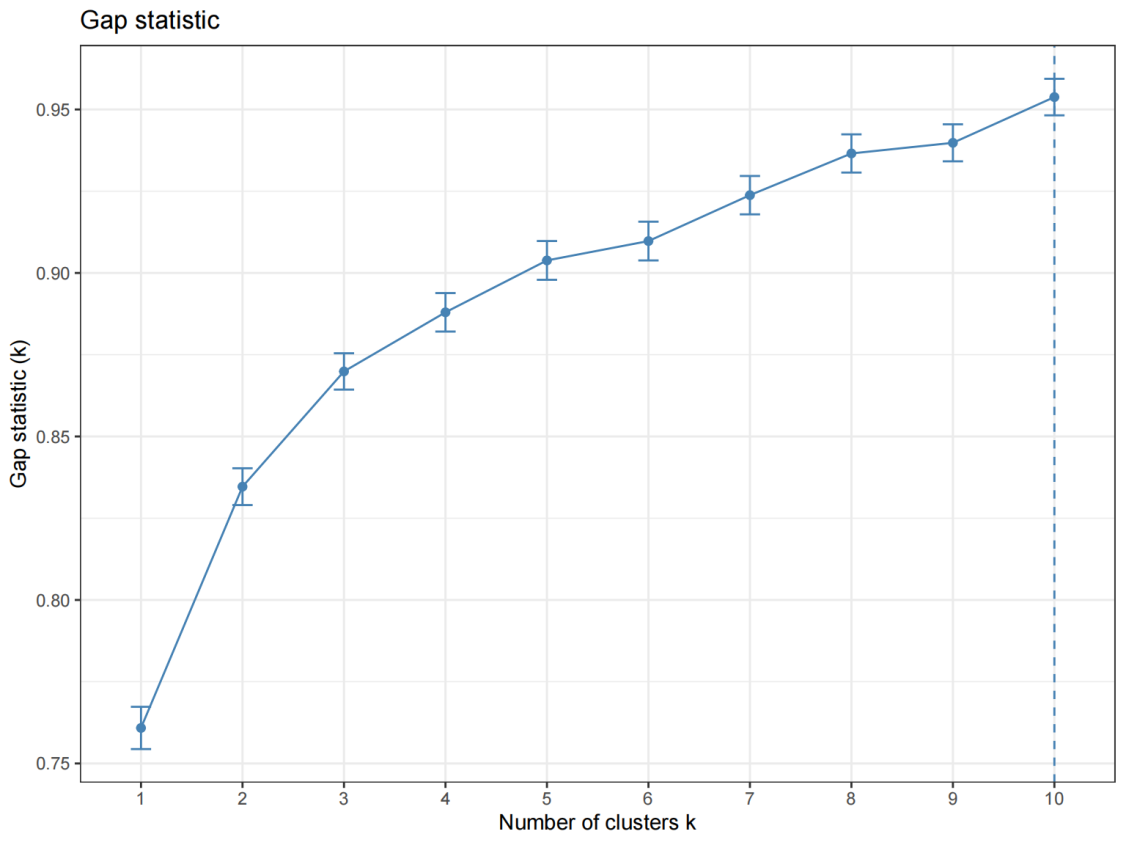


**Supplementary Figure 1.** Gap statistic for determining the optimal number of clusters. Gap statistics were calculated using the Tibshirani criterion. The gap statistic suggested a more granular partition, but k = 2 was selected as the primary working solution based on the combined evidence from the elbow method, silhouette width, hierarchical clustering validation, and clinical interpretability.


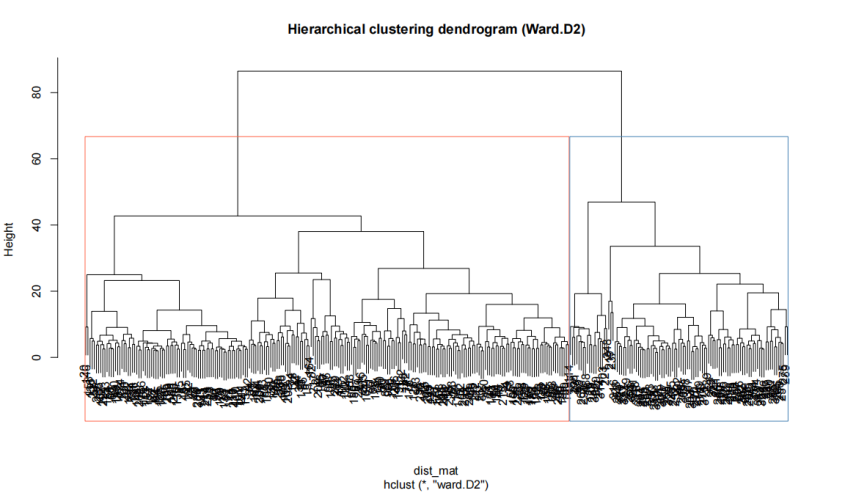


**Supplementary Figure 2.** Ward.D2 hierarchical clustering dendrogram of the standardized tongue features. Distances were calculated using Euclidean distance. Rectangles indicate the two-cluster cut. All continuous tongue features were z-score standardized before hierarchical clustering.


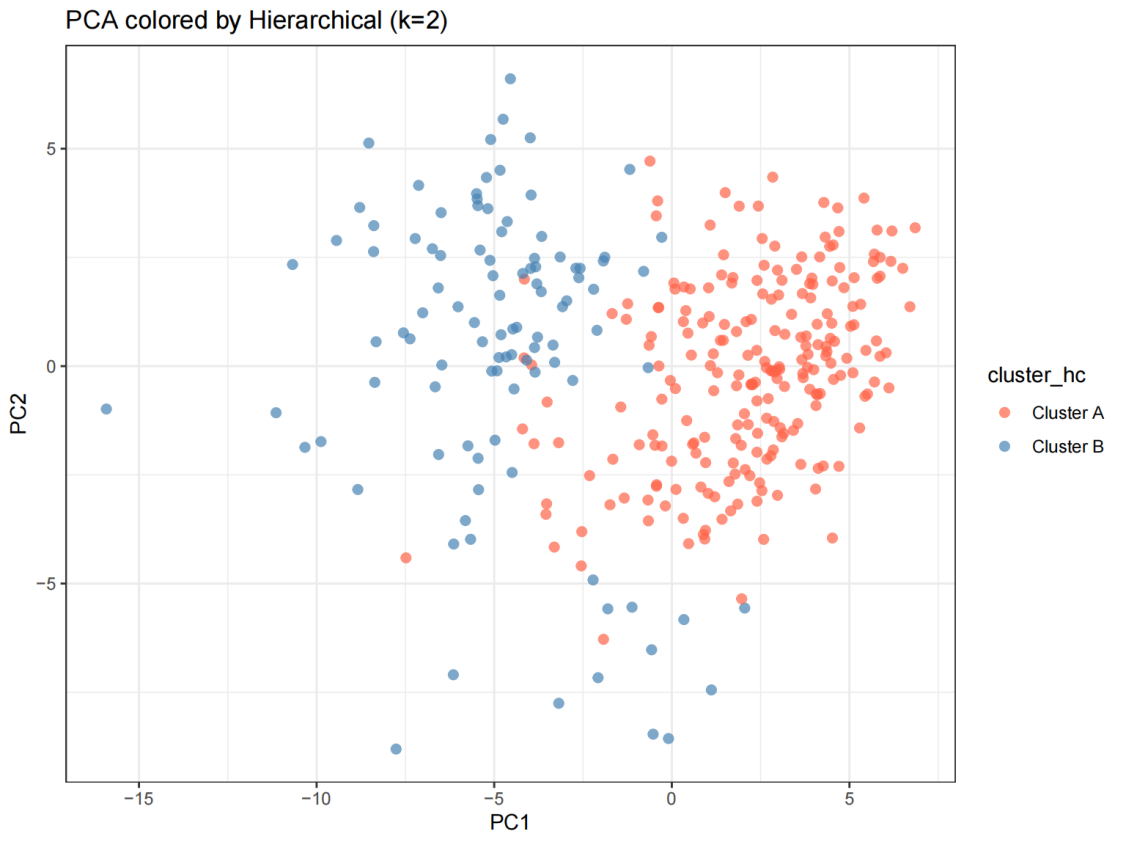


**Supplementary Figure 3.** PCA score plot colored by Ward.D2 hierarchical clustering assignment. The first two principal components explained 51.3% of the total variance. All continuous tongue features were z-score standardized before PCA. Clusters are colored according to the Ward.D2 hierarchical clustering solution at k = 2.


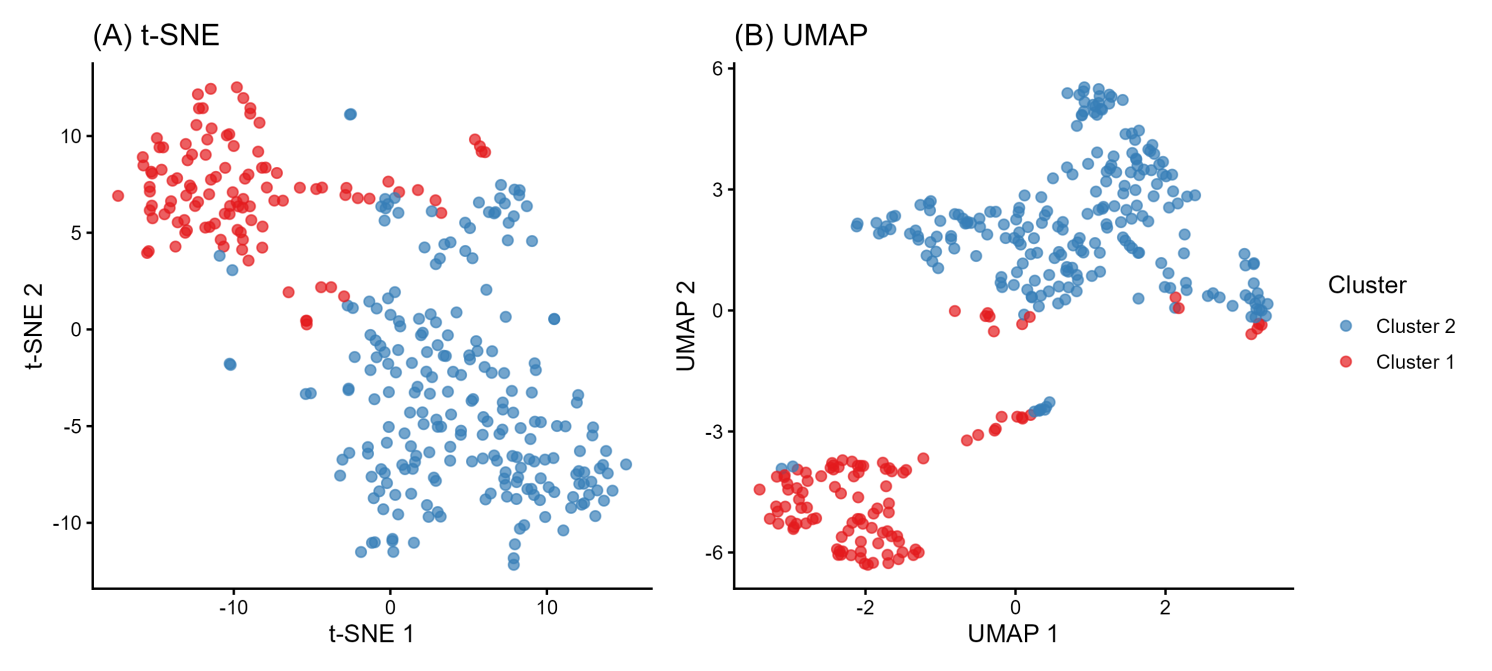


**Supplementary Figure 4.** t-SNE (A) and UMAP (B) projections of the z-score-standardized 48 continuous tongue features, colored by K-means cluster assignment at k = 2. Cluster 1 (red, n = 108) and Cluster 2 (blue, n = 223) show consistent spatial separation in both non-linear embeddings, with Cluster 1 forming a more compact distribution and Cluster 2 occupying a broader but largely distinct region. The UMAP projection reveals a more pronounced structural separation than the linear PCA score plot (Figure 1D), supporting the robustness of the identified two-cluster partition. t-SNE was computed with perplexity = 30 and 1,000 iterations; UMAP was run with default parameters (n_neighbors = 15, min_dist = 0.1). A fixed random seed was used for reproducibility.
